# Anti-inflammatory effect of glucose—mannose binding lectins isolated from Brazilian beans

**DOI:** 10.1080/09629359791695

**Published:** 1997-06

**Authors:** A. M. S. Assreuy, M. D. Shibuya, G. J. Martins, M. L. P. De Souza, B. S. Cavada, R. A. Moreira, J. T. A. Oliveira, R. A. Ribeiro, C. A. Flores

**Affiliations:** 1 Department of Physiology and Pharmacology Federal University of Ceara PO Box 3157 Rua Coronel Nunes de Melo, 1127 Fortaleza CE 60.430-270 Brazil; 2 Department of Biochemistry and Molecular Biology Federal University of Ceara Fortaleza CE Brazil

## Abstract

Selectins are essential for leukocyte recruitment in inflammation. Because of a lectin domain present in the selectin structure, we investigated the anti-inflammtory activity of six mannose–glucose binding lectins from brazilian beans: *Dioclea guianensis*-DguiL; *D. grandiflora*-DgL; *Cratylia floribunda*-CfL; *D. violacea*-D.vL; *D. virgata*-DvirL and *Canavalia brasiliensis*-ConBr. The lectins were injected intravenously (i.v.) into rats (0.1 and 1.0 mg/kg; 30 min before irritants) and its activities compared to *E. coli* endotoxin (LPS,30 μg/kg i.v.). Three lectins (DvL, CfL and DguiL), although less intense than LPS, inhibited the neutrophil migration induced by carrageenan (Cg, 300 μg) in a dose-dependent manner (0.1 and 1.0 mg/kg). DvL activity was reversed by 0.1 M α-D-methyl-mannoside (α-CH3), but not by 0.1 M α-D-galactose. The fMLP (44 ng)-induced neutrophil migration was also reduced by these lectins. Endotoxin contamination of lectin samples could be excluded since α-CH3 treatment reversed the DvL effect, but did not modify LPS inhibitory activity. Carrageenan (300 μg)-induced paw oedema was also reduced by LPS or lectin treatments. Conversely, none of the tested lectins inhibited dextran (Dex, 300 μg)-induced paw oedema, a classical leukocyte independent model, or zymosan (Zy, 1.0 mg)-induced peritonitis and paw oedema. LPS showed no effect upon Dex-induced paw oedema and barely reduced (25%) the oedematogenic effects of zymosan. As proposed for LPS, the lectin inhibitory activity was better observed on neutrophil-mediated inflammatory reactions. We speculate that the plant lectin antiinflammatory activity is probably due to a competitive blockage of a common leukocyte and/or endothelial selectin carbohydrate ligand.


Mediators of Inflammation, 6, 201–210 (1997)

### Anti-inflammatory effect of glucose–mannose binding lectins isolated from Brazilian beans

A. M. S. Assreuy,[1] M. D. Shibuya,[1] G. J. Martins,[1] M. L. P. de Souza,[1] B. S. Cavada,[2] R. A. Moreira,[2] J. T. A. Oliveira,[2] R. A. Ribeiro,[1,CA] C. A. Flores[1†]

[1]Department of Physiology and Pharmacology, Federal University of Ceara, PO Box 3157, Rua Coronel Nunes de Melo, 1127, 60.430-270 Fortaleza, CE, Brazil; [2]Department of Biochemistry and Molecular Biology, Federal University of Ceara, Fortaleza, CE, Brazil

[CA]Corresponding Author
Tel: (+55) 85 243 9349
Fax: (+55) 85 243 9333

† Dr C. A. Flores is now deceased.

**Selectins are essential for leukocyte recruitment in inflammation. Because of a lectin domain present in the selectin structure, we investigated the anti-inflammtory activity of six mannose–glucose binding lectins from brazilian beans: *Dioclea guianensis*-DguiL; *D. grandiflora*-DgL; *Cratylia floribunda*-CfL; *D. violacea*-D.vL; *D. virgata*-DvirL and *Canavalia brasiliensis*-ConBr. The lectins were injected intravenously (i.v.) into rats (0.1 and 1·0 mg/kg; 30 min before irritants) and its activities compared to *E. coli* endotoxin (LPS, 30 μg/kg i.v.). Three lectins (DvL, CfL and DguiL), although less intense than LPS, inhibited the neutrophil migration induced by carrageenan (Cg, 300 μg) in a dose-dependent manner (0.1 and 1·0 mg/kg). DvL activity was reversed by 0.1 M α-D-methyl-mannoside (α-$CH_3$), but not by 0.1 M α-D-galactose. The fMLP (44 ng)-induced neutrophil migration was also reduced by these lectins. Endotoxin contamination of lectin samples could be excluded since α-$CH_3$ treatment reversed the DvL effect, but did not modify LPS inhibitory activity. Carrageenan (300 μg)-induced paw oedema was also reduced by LPS or lectin treatments. Conversely, none of the tested lectins inhibited dextran (Dex, 300 μg)-induced paw oedema, a classical leukocyte independent model, or zymosan (Zy, 1.0 mg)-induced peritonitis and paw oedema. LPS showed no effect upon Dex-induced paw oedema and barely reduced (25%) the oedematogenic effects of zymosan. As proposed for LPS, the lectin inhibitory activity was better observed on neutrophil-mediated inflammatory reactions. We speculate that the plant lectin anti-inflammatory activity is probably due to a competitive blockage of a common leukocyte and/or endothelial selectin carbohydrate ligand.**

**Key words:** Anti-inflammatory, Mannose-binding plant lectins, Neutrophil migration, Rat paw oedema, Selectins

### Introduction

Neutrophil infiltration from the blood into the affected tissue is the hallmark of acute inflammatory reactions. The recruitment of these cells involves a complex and multi-mediated process which includes sequential interactions between these cells and endothelial cells and extracellular matrix components.[1–3] The control of these mechanisms involves both the activation of adhesion receptors already present in endothelial and resting blood cells, and the expression of new adhesion molecules on cell membranes. The crucial initial step of neutrophil migration depends on binding of the neutrophils to selectins expressed by both the above mentioned cells and venular endothelial cells activated by inflammatory mediators.[4] Three selectins, belonging to the C-type family of eukaryotic lectins, have been characterized.[5] The interactions involving selectins are dependent on the recognition of specific cell surface glycoconjugates.[6,7] For example, human endothelial P-selectin is able to specifically bind to sialylated, fucosylated polylactosamines on the surfaces of leukocytes.[8] A recent study has demonstrated that the lectin domain of two subunits of the B oligomer from pertussis toxin, S2 and S3, shares amino acid sequence similarity with the lectin domains of the eukaryotic selectin family.[9] Because selectins are essential for rolling of leukocytes on endothelial cells, in the inflammatory process, it was postulated that pertussis toxin could block leukocyte recruit-

© 1997 Rapid Science Publishers ment into inflamed tissue by competitive blockage of selectin binding sites. In fact, it has been demonstrated that S2 and S3 lectin domains or peptides derived from them competitively inhibit adherence of neutrophils to selectin-coated surfaces and to endothelial cells *in vitro*[10] as well as leukocyte recruitment into cerebrospinal fluid after pneumococcal challenge *in vivo*.[11] However, we have recently demonstrated that for the anti-inflammatory effect of pertussis toxin *in vivo*, the ADP-ribosilating activity of the A-protomer is more important than S2 and S3 lectin domains.[12,13] We have investigated the capacity of some plant lectins to antagonize, *in vivo*, the neutrophil migration induced by different inflammatory stimuli. Plant lectins can be defined as all plant proteins that possess at least one non-catalytic domain that binds reversibly to a specific mono- or oligosaccharide,[14] and are able to mediate several biological effects including histamine releasing properties,[15] human lymphocyte stimulation,[16] and, as the term lectin was initially referred, the ability to selectively agglutinate erythrocytes.[14] It is proposed that com- petitive activity between plant lectins and selectins by glycosylated binding sites present at the surface of the endothelium, leukocyte membranes and/or extracellular matrix components can expand the range of possibilities for development of selectin analogues with potential anti-inflammatory properties.

### Materials and Methods

### Animals

Male Wistar rats (150–250 g body weight) were housed in a temperature-controlled room with free access to water and food.

### Lectins

Lectins from six leguminous seeds from the *Dioclinae* subtribe (family *Leguminosae*, tribe *Phaseolae*) were studied. Lectin isolation by affinity chromatography on Sephadex G-50 has been previously described. *Dioclea guianensis*,[17] *Dioclea grandiflora*,[18] *Cratylia floribunda*,[19] *Dioclea violacea*,[20], *Dioclea virgata*,[21] and *Canavalia brasiliensis*.[22] All lectins used in this study are of the glucose–mannose type.

### Neutrophil migration into peritoneal cavity of rats

Carrageenan (300 μg), zymosan (1 mg) or fMLP (44 ng) were injected intraperitoneally (i.p.) in 1 ml of sterile 0.15 M NaCl. After 2–8 h, animals were sacrificed and peritoneal lavage was performed with 10 ml of sterile phosphate buffered saline (PBS) containing 5 U/ml heparin. The fluid was removed for total and differential cell counts and results were reported as mean ± SEM of the number of cells per microlitre of peritoneal wash.[23] Lectins (0.1 and 1·0 mg/kg), alone or in combination with 0.1 M of α-D-methyl-mannoside or α-D-galactose were injected i.v. into rats 30 min before the inflammatory stimuli (0·1 ml/100 g body weight). LPS (30 μg/kg, i.v.) was used as positive control for the inhibitory effect on neutrophil migration. All drugs were dissolved in sterile 0.15 M NaCl. Control animals received sterile saline i.v. (0·1 mg/100 g body weight).

### Inflammatory paw oedema

Paw oedema was induced by subplantar injection of carrageenan (300 μg/paw), zymosan (1 mg/paw) or dextran (300 μg/paw) in a final volume of 0.1 ml into the right hind paw of rats under light ether anaesthesia. All drugs were dissolved in sterile 0.15 M NaCl. Paw volume was measured immediately before the irritant injections, and at selected time intervals thereafter with a hydroplethysmometer.[24] LPS (30 μg/kg) or lectins (1 mg/kg) were injected i.v. 30 min before injection of the irritants. Results were expressed as the increase in paw volume (ml) calculated by subtracting the basal volume. The area under the time-course curve was also calculated using a trapezoidal rule and results expressed in arbitrary units.[24]

### Haemagglutinating activity

Clumping by the six purified lectins of a 2% rat erythrocyte suspension in 0.15 M NaCl containing 5 mM $CaCl_2$ and $MnCl_2$ was estimated as described elsewhere.[25] Haemagglutinating activity was expressed as haemagglutinating units (HU)/ml. One HU was defined as the inverse of the end point of a serial dilution of an initial lectin solution (2 mg/ml) required to induce visible agglutination. Determinations were made in duplicate. We also compared this activity using erythrocytes from other rodent species, e.g. rabbit and mouse.

### Drugs

The following drugs were used: carrageenin (BDH Chemicals, UK), zymosan (Sigma, USA), *N*-formyl-L-methionyl-L-leucyl-L-phenylalanine, (fMLP; Sigma, USA), Dextran 70 (Pharmacia,

USA), LPS from *E. coli* 011:B4 (Difco, USA), α-D-methyl mannoside (Sigma, USA) and α-D-galatose (Merck, Germany). All other chemicals were analytical grade preparations.

### Statistical analysis

All results were expressed as the mean ± SEM for n experiments with the exception of haemaglutinating activity which was expressed in median values. Statistical evaluation was undertaken by analysis of variance (ANOVA) and Duncan's test for multiple comparison. A *P*-value of less than 0.05 was considered significant.

### Results

### Screening for inhibitory activity of six glucose–mannose type lectins on carrageenan-induced neutrophil migration

Carrageenan (Cg, 300 μg/cavity) caused a significant ($P < 0{\cdot}05$) neutrophil migration when injected into the peritoneal cavities of rats (Fig. 1 and Fig. 3). Among the lectins tested, three inhibited the neutrophil migration induced by Cg in a dose-dependent manner: *D. violacea* lectin caused reductions of 29 and 70%, *Cratylia floribunda* lectin caused 29 and 62% reductions and *D. guianensis* lectin caused 43 and 63% reductions in inhibition at 0.1 and 1·0 mg/kg, respectively, measured 4 h after the inflammatory challenge (Fig. 1A–C). *D. virgata* lectin also decreased the Cg-induced neutrophil infiltration (54 and 40% reductions), but not in a dose-dependent manner (Fig. 1D). On the other hand, *D. grandiflora* and *Canavalia brasiliensis* lectins, although belonging to the same group of lectins, did not alter the number of infiltrating neutrophils, when compared to the saline-injected (i.v.) animals (SAL group), at either dose tested (Fig. 1E and 1F). However, the effect of those inhibitory lectins was less intense than that promoted by LPS (30 μg/kg), which reached 93% of inhibition, and was used in this work as positive control. At this dose LPS produces maximal inhibitory effect in this model,[26] and this activity could not be accounted for by hypotension.[27]

### Effect of lectins from *Cratylia floribunda, D. guianensis* and *D. violacea* on neutrophil migration induced by fMLP and zymosan fMLP (44 ng, i.p.) and zymosan (1 mg, i.p.) caused a significant ($P < 0{\cdot}05$) neutrophil migration into the peritoneal cavity when measured 4 h after the inflammatory challenge (Fig. 2). Treatment of animals, 30 min before the inflammatory stimuli, with 1 mg/kg (i.v.) of *Cratylia floribunda*, *D. guianensis* or *D. violacea* lectins strongly reduced ($P < 0{\cdot}05$) the fMLP-induced neutrophil migration, by 73, 84 and 100%, respectively (Fig. 2A). Conversely, none of these lectins inhibited zymosan-induced neutrophil migration (Fig. 2B). The neutrophil migration induced by fMLP or zymosan was strongly inhibited by 30 μg/kg of LPS ($P < 0{\cdot}05$).

### *D. violacea* seed lectin effect upon carrageenan(Cg)- and fMLP-induced neutrophil migration and the involvement of carbohydrate residues on its inhibitory activity

*D. violacea* lectin was used to investigate the involvement of mannose and glucose residues as a site for the inhibitory effect of lectins. This lectin was chosen because it showed the highest activity at 1·0 mg/kg. At this dose, the lectin from *D. violacea*, when injected 30 min before Cg inhibited the neutrophil migration seen 4 and 8 h following i.p. administered Cg (Fig. 3A). The i.v. co-injection of 1·0 mg/kg of this lectin with 0.1 M of α-D-methyl-mannoside (one specific binding sugar), 30 min before Cg (300 μg, i.p.; Fig. 3B) or fMLP (44 ng, i.p.; Fig. 4), blocked ($P < 0{\cdot}05$) its inhibitory effect. On the other hand, α-D-galactose (0·1 M) did not change the inhibitory activity of *D. violacea* lectin on Cg-induced neutrophil migration (Fig. 3B). Co-injection of LPS (30 μg/kg) with 0.1 M of α-methyl-mannoside did not change the LPS inhibitory effect on the fMLP-induced neutrophil migration model (Fig. 4). Thus, we can rule out the possibility that the *D. violacea* lectin activity was caused by endotoxin contamination.

### Effect of LPS and plant lectins on rat paw oedema induced by carrageenan (Cg), zymosan and dextran

Subplantar injection of Cg (300 μg/paw) induced a progressive and intense paw oedema that reached a maximal value by 3 h (Fig. 5A). Lectins from *D. violacea*, *Cratylia floribunda* or *D. guianensis* seeds, injected i.v. at 1 mg/kg, 30 min before the chemotactic agent, reduced the Cg-induced paw oedema by 32, 30 and 17%, respectively. LPS (30 μg/kg) treatment caused a 50% reduction in the paw oedema induced by Cg (Fig. 5B). The inhibitory effect of the *D.*

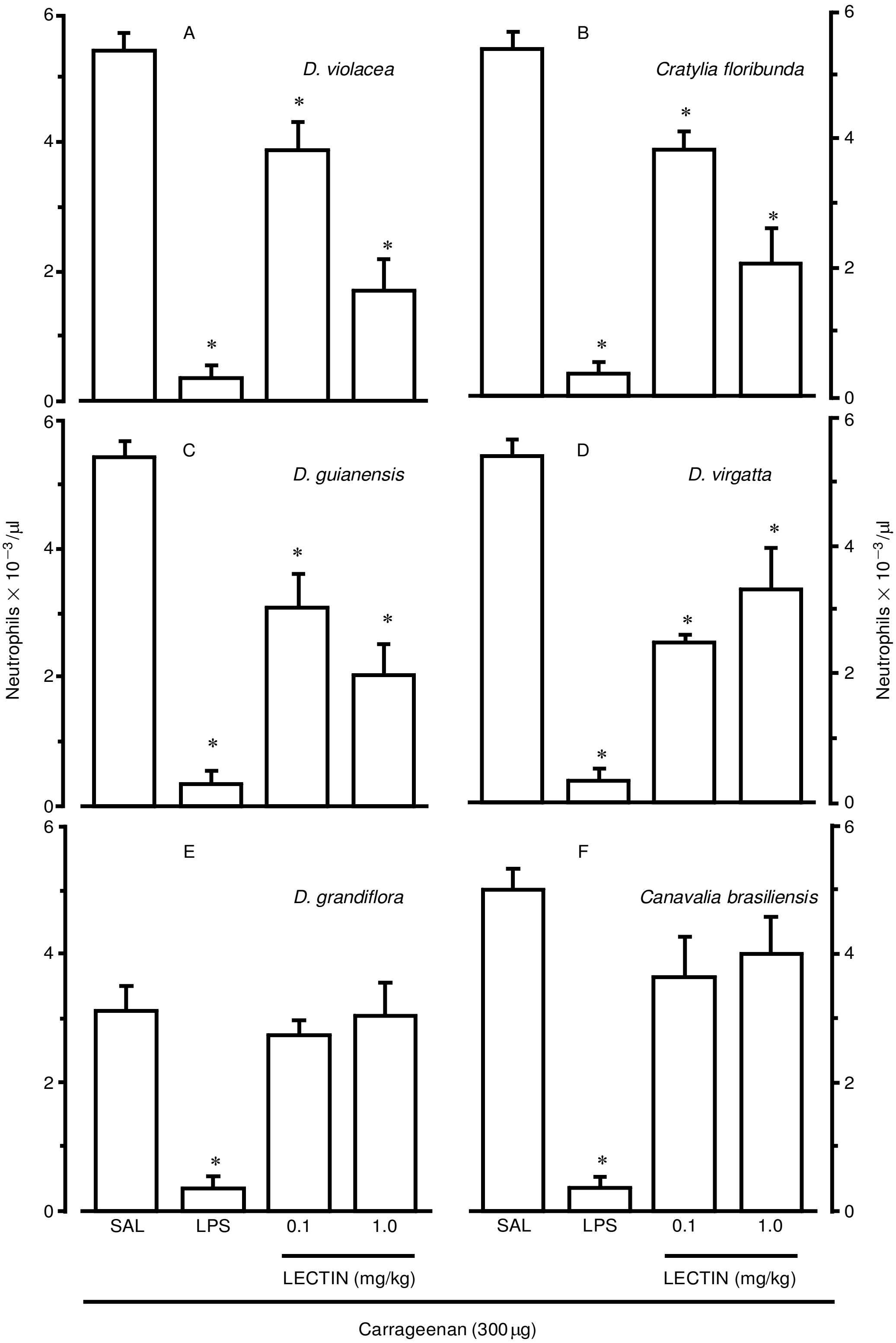

FIG. 1. Effect of i.v. injection of *E. coli* endotoxin (LPS) and lectins from *D. violacea* (A), *Cratylia floribunda* (B), *D. guianensis* (C), *D. virgata* (D), *D. grandiflora* (E) and *Canavalia brasiliensis* (F) seeds upon neutrophil migration into rat peritoneal cavities induced by carrageenan (Cg, 300 µg). The animals were treated 30 min before the inflammatory stimuli, i.v. (0·1 ml/100 g), with saline (SAL), LPS (30 µg/kg) or lectins (0.1 and 1·0 mg/kg). The migration was evaluated 4 h after Cg injection. Values are mean ± SEM for the number of animals used (n = 5–11). The number of neutrophils present 4 h after i.p. saline injection was 0·47 × $10^{-3}$ ± 0·13/µl (n = 6). Asterisks indicate a significant statistical difference ($P < 0.05$, ANOVA–Duncan's test), compared to the SAL control group.

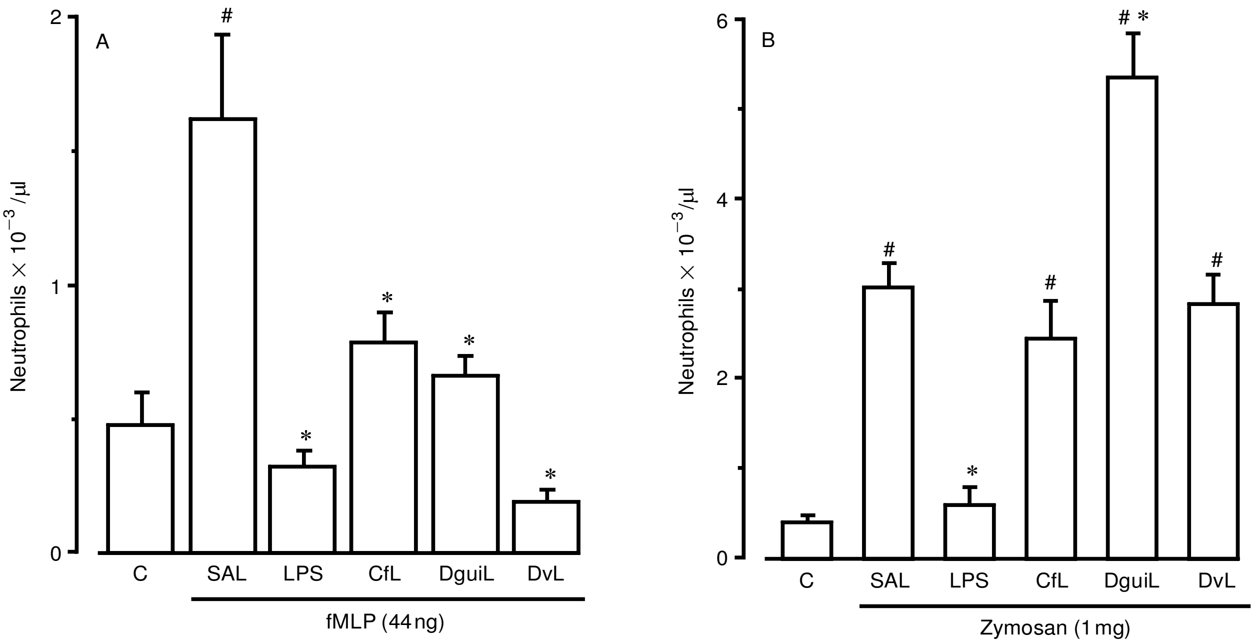

FIG. 2. Effect of i.v. injection of *E. coli* endotoxin (LPS) and lectins from *Cratylia floribunda* (CfL), *D. guianensis* (DguiL) and *D. violacea* (DvL) seeds on neutrophil migration into the peritoneal cavity in rats injected with fMLP (44 ng, A) or zymosan (1 mg, B). The lectins (1 mg/kg, i.v.) and LPS (30 µg/kg) were administered 30 min before the inflammatory stimulus. Infiltrates for cell counts were collected 4 h after fMLP or zymosan injections. Control animals (C) received saline (0·1 ml/100 g) both by i.v. and i.p. routes. SAL refers to animals that received saline i.v. and Cg i.p. Values are mean ± SEM for the number of animals used (n = 5–12). * Indicates a significant statistical difference when compared to the SAL group ($P < 0.05$); # indicates a significant statistical difference when compared to the C group ($P < 0.05$) (ANOVA, Duncan's test).

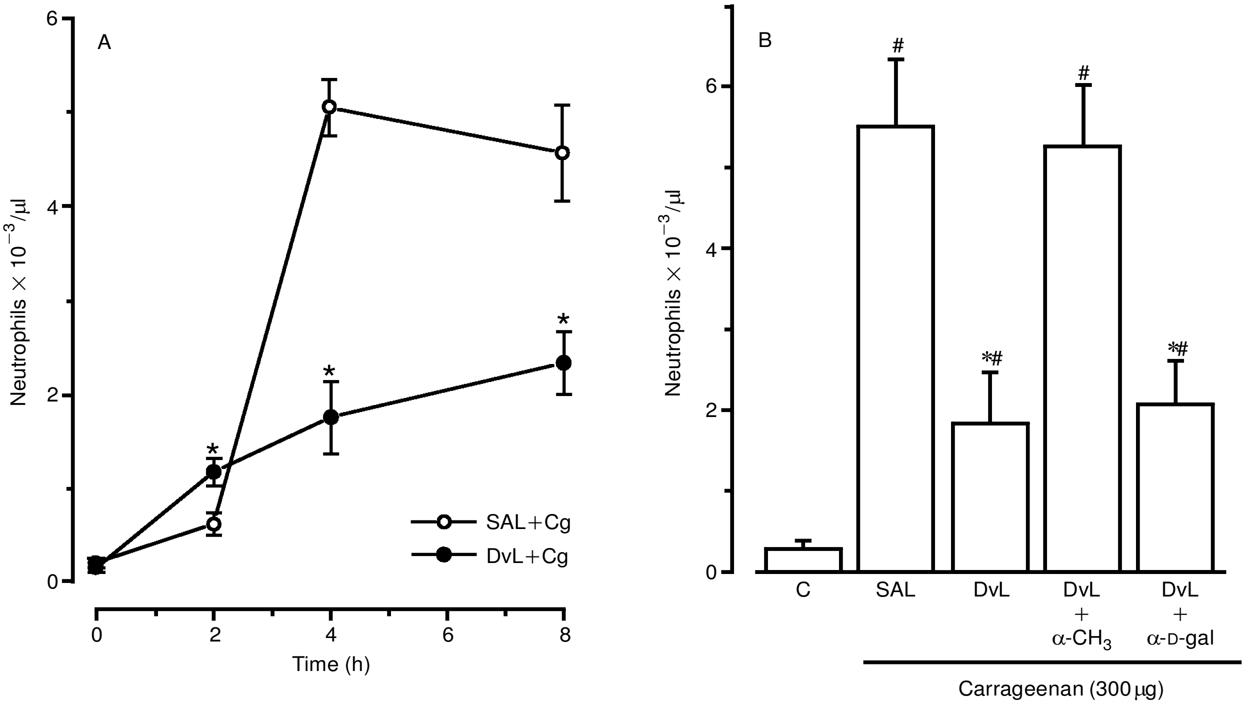

FIG. 3. Effect of *D. violacea* seed lectin (DvL) on carrageenan (Cg)-induced neutrophil migration into rat peritoneal cavities and the involvement of mannose/glucose residues on its inhibitory activity. (A) Time-course of Cg-induced neutrophil migration and the effect of DvL treatment (1·0 mg/kg in 0·1 ml/100 g, i.v. 30 min before i.p. injection of 300 µg Cg). (B) The effect of 0.1 M α-D-methyl-mannoside (α-$CH_3$) or α-D-galactose (α-D-gal) in DvL inhibitory activity, 4 h after Cg-challenge. Control animals (C) received saline (0·1 ml/100 g) both by i.v. and i.p. routes. SAL refers to animals that received saline i.v. and carrageenin i.p. Values are mean ± SEM for the number of animals used (n = 6). * Indicates a significant statistical difference when compared to the SAL group ($P < 0.05$); # indicates a significant statistical difference when compared to the C group ($P < 0.05$) (ANOVA, Duncan's test).

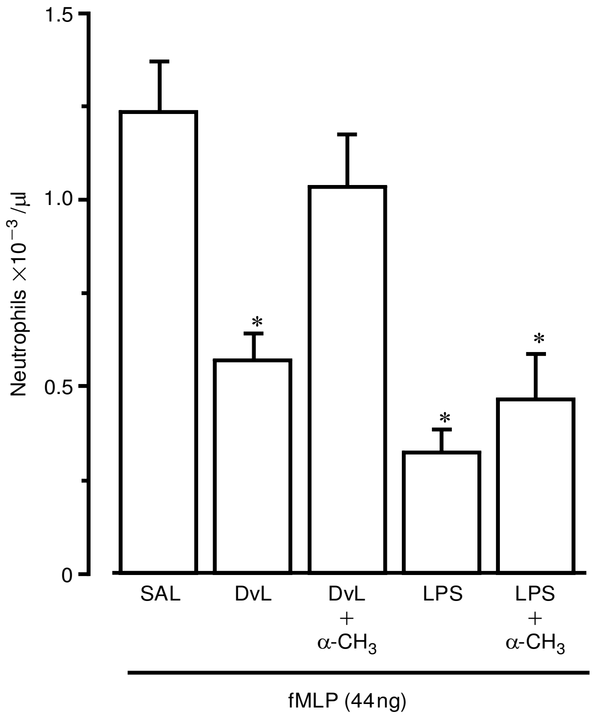

FIG. 4. The inhibitory effect of *D. violaceae* lectin (DvL) on fMLP-induced neutrophil migration is not due to endotoxin contamination. DvL (1·0 mg/kg) or LPS (30 µg/kg) were injected i.v. alone or in combination with α-$CH_3$ (0·1 M) 30 min before i.p. injection of fMLP (44 ng). The cell counts were made 4 h after the inflammatory stimulus challenge. SAL refers to animals that received saline intravenously and fMLP intraperitoneally. Values are mean ± SEM for the number of animals used (n = 5–10). * Indicates a significant statistical difference ($P < 0.05$) compared to the SAL group (ANOVA, Duncan's test).

*violacea* lectin was blocked ($P < 0{\cdot}05$) by co-injection with 0.1 M of α-D-methyl-mannoside (Fig. 6). On the other hand, 0.1 M of α-D-galactose did not alter the activity of the *D. violacea* lectin. In this set of experiments, paw oedema values 4 h after Cg challenge were (ml, mean ± SEM): *D. violacea* + Cg = 0·44 ± 0.06, n = 5; *D. violacea* + α-D-galactose + Cg = 0·51 = ±0.05, n = 6; saline + Cg = 0·65 ± 0·003, n = 5. Dextran (300 µg/paw) and zymosan (1 mg/paw) induced a greater paw oedema with a more rapid onset; it peaked at 1 h and remained elevated up to 4 h after the inflammatory stimuli. In contrast to the effect observed on Cg-induced paw oedema, none of the tested lectins inhibited the oedema development induced by dextran (Table 1) or zymosan (Table 2). LPS (30 µg/kg) showed no effect upon dextran-induced paw oedema (Table 1) and reduced the zymosan oedematogenic effect by only 25% (Table 2).

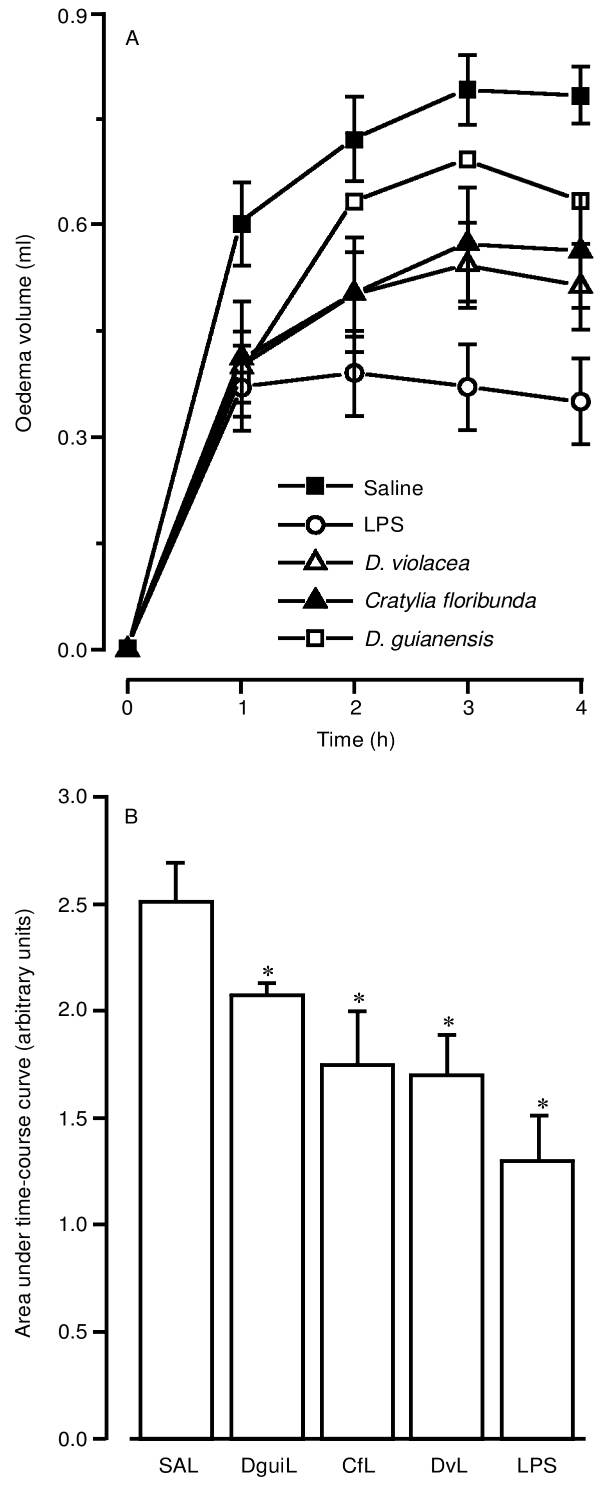

FIG. 5. Inhibition by lectins from *D. violacea* (DvL), *Cratylia floribunda* (CfL) and *D. guianensis* (DguiL) of rat paw oedema induced by intraplantar injection of carrageenan (300 µg/0·1 ml). The animals were treated (0·1 ml/100 g, i.v.) 30 min before Cg injection, with saline (SAL), LPS (30 µg/kg) or lectins (1 mg/kg). Control animals received Cg both by intraplantar and i.v. routes. (A) Oedema was measured at 1, 2, 3 and 4 h after the inflammatory challenge and expressed as the increase in paw volume (ml) above its basal volume. (B) The area under the time-course curve was determined using a trapezoidal rule. Each point represents the mean ± SEM from six rats. * Indicates a significant statistical difference ($P < 0.05$), compared to the SAL group (ANOVA, Duncan's test).

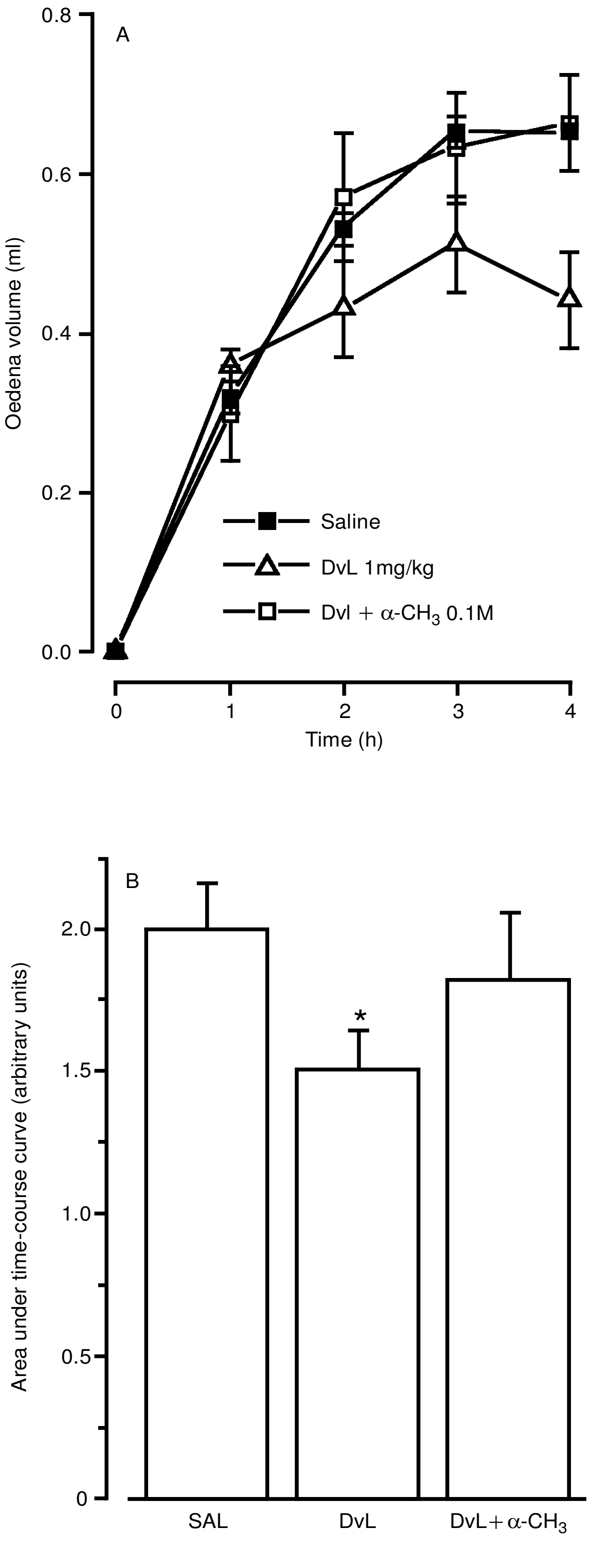

FIG. 6. α-D-methyl-mannoside (α-$CH_3$, 0·1 M) blocks the inhibitory effect of *D. violacea* (DvL) on the carrageenan (Cg)-induced rat paw oedema. The animals received saline, DvL or DvL + α-$CH_3$ 30 min before Cg (300 μg/paw). (A) Oedema was measured at 1, 2, 3 and 4 h after the inflammatory challenge and expressed as the increase in paw volume (ml) above its basal volume. (B) The area under the time-course curve was determined using a trapezoidal rule. Each point represents the mean ± SEM from 5–10 rats. * Indicates a significant statistical difference ($P < 0.05$), compared to the SAL group (ANOVA, Duncan's test).

### Haemagglutinating activity of lectins in rodent erythrocytes

Haemmagglutinating activity of lectins from *D. violacea*, *Cratylia floribunda*, *D. guianensis*, *D. virgata*, *D. brasiliensis* and *D. grandiflora* seeds was estimated on a 2% suspension of rat, rabbit or mouse erythocytes; haemagglutinating activity differed greatly among animal species. In general, the highest susceptibility to lectin haemagglutinating activity was shown by rabbit erythrocytes. For example, in these cells the lectin haemagglutinating activity of *Cratylia floribunda* was 64-fold higher than that observed for rat erythrocytes. Furthermore, *Cratylia floribunda* and *D. violacea* lectins which showed the highest anti-inflammatory activity (Fig. 1), showed the lowest HU in rat erythrocytes (Table 3).

### Discussion

We have demonstrated that some mannose–glucose binding lectins from seeds of the *Dioclinae* show anti-inflammatory activity. Similar to that observed with LPS, the i.v. administration of lectins from *D. violacea*, *D. guianensis*, *Cratylia floribunda* and *D. virgata* seeds inhibited both the neutrophil migration into rat peritoneal cavities induced by fMLP or carrageenan, and rat hind paw oedema induced by carrageenan. Among the lectins tested the highest anti-inflammatory activity was shown by *D. violacea* lectin (DvL), the anti-inflammatory effect of which could be blocked by α-D-methyl-mannoside (a specific lectin binding sugar) but not by α-D-galactose. When administered alone these sugars showed no anti-inflammatory activity (data not shown); this is in accordance with the demonstration that D-mannose had no significant effect on the neutrophil migration induced *in vivo* or *in vitro* by fMLP.[28] Furthermore, α-D-methyl-mannoside did not modify the LPS inhibitory effect on fMLP-induced neutrophil migration showing that DvL activity was not due to endotoxin contamination. These data are in favour of a specific lectin activity. However, *D. grandiflora* and *Canavalia brasiliensis* seed lectins, although belonging to the same mannose–glucose binding group, were devoid of any inhibitory activity. In addition, we also observed that concanacalin A, a well-known member of this group of plant lectins, did not inhibit fMLP-induced neutrophil migration (data not shown), in spite of the demonstration of its immunosupressive effect,[29] and inhibitory activity on experimental allergic encephalomyelitis

**Table 1**. Effect of plant lectins and LPS on the time course of paw oedema induced in rats by dextran

| | Time (hours) | | | | |
|---|---|---|---|---|---|
| Treatment[a] | 0.5 | 1.0 | 2.0 | 3.0 | n[b] |
| Saline | 0.83 ± 0.05[c] | 0.88 ± 0.07 | 0.79 ± 0.05 | 0.72 ± 0.06 | 22 |
| LPS | 0.91 ± 0.08 | 0.89 ± 0.06 | 0.79 ± 0.07 | 0.60 ± 0.11 | 6 |
| Lectins | | | | | |
| DvL[d] | 0.98 ± 0.04 | 1.04 ± 0.06 | 0.92 ± 0.04 | 0.81 ± 0.05 | 6 |
| DguiL[d] | 0.84 ± 0.04 | 0.97 ± 0.06 | 0.89 ± 0.07 | 0.79 ± 0.07 | 6 |
| CfL[d] | 0.89 ± 0.10 | 0.85 ± 0.07 | 0.74 ± 0.09 | 0.71 ± 0.10 | 4 |

[a]Lectins (1 mg/kg) or LPS (30 μg/kg) were injected i.v. 30 min before dextran (300 μg/0·1 ml, intraplantar route). The control group received saline (0·1 ml/100 g weight).
[b]n, number of animals/group.
[c]Results are expressed as the mean ± SEM of the increase in paw volume (ml).
[d]DvL, *Dioclea violacea*; CfL, *Cratylia floribunda*; DguiL, *Dioclea guianensis*.

**Table 2**. Effect of plant lectins and LPS on the time course of paw oedema induced in rats by zymosan

| | Time (hours) | | | | |
|---|---|---|---|---|---|
| Treatment[a] | 1 | 2 | 3 | 4 | n[b] |
| Saline | 0.94 ± 0.04[c] | 0.96 ± 0.04 | 0.95 ± 0.03 | 0.90 ± 0.03 | 22 |
| LPS | 0.85 ± 0.05 | 0.76 ± 0.05[e] | 0.77 ± 0.03[e] | 0.65 ± 0.03[e] | 12 |
| Lectins | | | | | |
| DvL[d] | 1.07 ± 0.06 | 1.01 ± 0.02 | 0.94 ± 0.06 | 0.92 ± 0.04 | 6 |
| CfL[d] | 1.01 ± 0.04 | 1.05 ± 0.02 | 0.97 ± 0.02 | 0.99 ± 0.05 | 6 |
| DguiL[d] | 0.82 ± 0.10 | 0.98 ± 0.07 | 0.94 ± 0.09 | 0.81 ± 0.09 | 6 |

[a]Lectins (1 mg/kg) or LPS (30 μg/kg) were injected i.v. 30 min before zymosan (1·0 mg/0·1 ml, intraplantar route). The control group received saline (0·1 ml/100 g weight).
[b]n, number of animals/group.
[c]Results expressed as mean ± SEM of increase in paw volume (ml).
[d]DvL = *Dioclea violacea*; CfL = *Cratylia floribunda*; DguiL = *Dioclea guianensis*.
[e]$P < 0.05$ (Anova, Duncan's test).

**Table 3**. Hemagglutinating activity of plant lectins on rodent erythrocytes

| Plants | Hemagglutinating units (HU)[a] | | |
|---|---|---|---|
| | Rat | Mouse | Rabbit |
| *Dioclea violacea* | 768[b] | 3072 | 3072 |
| | (64–8192) | (512–32 768) | (256–8192) |
| *Cratylia floribunda* | 512 | 6144 | 32 768 |
| | (256–16 384) | (2048–32 768) | (8192–32 768) |
| *Canavalia brasiliensis* | 1024 | 768 | 16 384[c] |
| | (512–2048) | (128–8192) | (8192–32 768) |
| *Dioclea grandiflora* | 2048 | 1024 | 2560[c] |
| | (512–4096) | (512–8192) | (512–8192) |
| *Dioclea guianensis* | 2048 | 1536 | 8192 |
| | (1024–4096) | (512–4096) | (4096–16 384) |
| *Dioclea virgata* | 12 288 | 32 768 | 24 576[c] |
| | (512–32 768) | (512–32 768) | (8192–32 768) |

[a]According to Moreira and Perroni.[25] The haemagglutinating unit (HU) was defined as the reciprocal of the highest serial lectin dilution (initial lectin solution 2 mg/ml) able to agglutinate a 2% erythrocyte suspension in 0.15 M NaCl with 5 mM $CaCl_2$ and $MnCl_2$.
[b]Results expressed as median of three experiments performed in duplicate. The numbers in parentheses show the range.
[c]Two experiments performed in duplicate.

in guinea pigs and rats.[30] Discrepancies in the capacity of mannose binding plant lectins to induce human lymphocyte stimulation, interferon-gamma release by human peripheral mononuclear leukocytes,[16] and rat mast cell degranulation,[15] were also observed. Interestingly, the lectins from the *Dioclinae* subtribe plants show very high homology (80–90%) with respect to their primary structures.[31] It has been suggested that despite very similar physico-chemical properties, differences in the biological activities of lectins might be due to differences in its fine sugar affinity sites that bind to target effectors.[15] Very recently, it was demonstrated that the crystal structure of *Canavalia brasiliensis* lectin suggests a correlation between its quaternary conformation and its biological properties distinct from those of concanavalin A.[32] Our results suggest that the lectin inhibitory activity could be better observed on neutrophil-mediated inflammatory reactions, since all lectins tested, like LPS, had no effect on dextran-induced paw oedema, a classical neutrophil-independent inflammatory agent.[33] The exact mechanism involved in the anti-inflamamtory effect of the studied plant lectins is currently unknown. The amount of lectin injected was in excess of the amount required for haemagglutination. However, no clear relationship could be demonstrated between lectin haemaglutinating and anti-inflammatory activities in the rat. DvL and *Cratylia floribunda* lectin showed the lowest haemagglutination activity and the highest anti-inflammatory activities. Beside this, *D. grandiflora* and *D. guianensis* lectins exhibited similar haemaglutinating activities but only the latter showed anti-inflammatory activity. Moreover, no alterations could be detected in the haematocrit of rats treated i.v. with Dvl compared to normal values (data not shown); no signs of acute toxicity were observed after i.v. administration of lectins, even in doses up to 5- to 10-fold higher than the maximal dose used in this study (data not shown), a finding which parallels previous results showing that concanavalin A, at similar dose range, did not cause acute toxicity.[29,30] Furthermore, the lectin inhibitory activity could not be accounted for by a possible leukopaenic or neutropenic effect, since none of the lectins tested changed, during the first 4 h, the numbers of circulating leukocytes when compared to saline-treated rats (data not shown). We speculate that these plant lectins are operating against the adhesive activities or selectins, competing with these glycoproteins by carbohydrate ligands on endothelial or leukocyte cell membranes. Which selectin is competitively blocked by these mannose binding lectins is unclear. The leukocyte adhesion mediated by selectins can be reduced by a variety of simple and complex carbohydrates, most of which are sialylated, fucosylated, or both.[5] In humans, the most important sialylated glycoconjugate is the sialosyl-Lewis-X which is expressed in large amounts by neutrophils and is considered the major binding site for selectins.[5] However, since this epitope is highly species specific and is absent in non-human mammalian species it cannot be considered to be a general binding site for interaction with selectins in non-human leukocytes.[34,13] As the lectin domain of E-selectin aligns closely with that of the mannose binding C-type lectins,[5] we postulated that it would be considered as the first candidate to compete with mannose binding plant lectins by the same carbohydrate ligands on neutrophil membranes. Corroborating this suggestion, we showed that plant lectin inhibitory activity was not detectable at 2 h after i.p. carrageenan injection, and it has been demonstrated that E-selectin plays essentially no role in the earliest phases (up to 2 h) of leukocyte migration during acute inflammation, because it requires *de novo* gene transcription.[5] In addition, E-selectin expression by cultured human endothelial cell peaks between 3 and 6 h after TNF-α stimulation,[35] a similar time interval for maximal neutrophil recruitment response following carrageenan injection into the rat peritoneal cavity. The ability of carrageenan to induce TNF-α release, in rats, has also been suggested.[36] However, it must be pointed out that not all neutrophil-mediated inflammatory reactions could be inhibited by these plant lectins. It was observed that lectins were ineffective in inhibiting both neutrophil migration and paw oedema induced by zymosan. These contradictory findings are difficult to explain. These data, however, ruled out the possibility that the observed anti-inflammatory activity of the mannose binding plant lectins could be due to a non-specific effect, such as hypotension or leukopaenia. Furthermore, as zymosan-induced neutrophil migration was unchanged or potentiated by plant lectins, a direct inhibitory effect upon neutrophils is probably not occurring. The possibility that the time interval we choose to test the lectin inhibitory activity was different from that reported for maximal zymosan-induced intraperitoneal neutrophil migration in mice[37] is unlikely, since, in rats, the maximal response occurred 4 h after peritoneal zymosan challenge (data not shown). It is possible, however, that zymosan triggers different neutrophil recruitment mechanisms, not sensitive to plant lectin treatment; zymosan is a well-known powerful agent in inducing the release of oxygen free radicals by macrophages,[37] the predominant peritoneal resident cell in rats, and it has been shown that hydrogen peroxide is able to induce long-lasting P-selectin expression[38] when compared to that induced by thrombin, histamine or complement fragments.[5] Thus we speculate that in zymosan-inflamed rat peritoneum P-selectin could play a pivotal role, and that P-selectin ligands, such as P-selectin glycoprotein ligand-1,[39] are probably not recognized by these mannose binding plant lectins.

In conclusion, the present work shows that mannose binding plant lectins can reduce neutrophil-mediated inflammatory reactions induced by different stimuli, such as carrageenan and fMLP. We suggest that lectin anti-inflammatory activity involves competition between plant lectins and selectins for a common carbohydrate ligand present on leukocyte and/or endothelial cell membranes. This hypothesis reinforces the proposition by others that sugar-based inhibitors directed against adhesive activities of selectins might provide for new and more effective anti-inflammatory drugs.[40,10,11] Thus, plant lectins could be used as important and specific tools for better understanding the role of sugar residues in leukocyte recruitment, as well as a template in the study of molecular interactions between selectins and their carbohydrate ligands.

### References

1. McEver RP. Leucocyte-endothelial cell interactions. *Current Biol* 1992; **4**: 840–849.
2. Varki A. Selectins and other mammalian sialic-acid binding lectins. *Current Opin Cell Biol* 1992; **4**: 257–266.
3. Cronstein BN, Weissmann G. The adhesion molecules of inflammation. *Arthritis Rheumatism* 1993; **36**: 147–157.
4. Frenette PS, Wagner DD. Adhesion Molecules – Part II: Blood vessels and blood cells. *New Eng J Med* 1996; **335**: 43–45.
5. Kansas GS. Selectins and their ligands: Current concepts and controversies. *Blood* 1996; **88**: 3259–3287.
6. Lasky LA. Selectins: Interpreters of cell-specific carbohydrate information during inflammation. *Science* 1992; **258**: 964–969.
7. Tedder TF, Steeber DA, Chen A, Engel P. The selectins: vascular adhesion molecules. *FASEB J* 1995; **9**: 866–873.
8. Tuomanen E. Subversion of leukocyte adhesion systems by respiratory pathogens. *Am Soc Microbiol* 1993; **59**: 292–295.
9. Saukkonen K, Burnette WN, Mar V, Masure HR, Tuomanen E. Pertussis toxin has eukaryotic like carbohydrate recognition domains. *Proc Natl Acad Sci USA* 1992; **89**: 118–122.
10. Rozdzinsk E, Burnette WN, Jones T, Mar V, Tuomanen E. Prokaryotic peptides that block leukocyte adherence to selectins. *J Exp Medicine* 1993; **178**: 917–924.
11. Rozdzinsk E, Jones T, Burnette WN, Burroughs M, Tuomanen E. Anti-inflammatory effects in experimental meningitis of prokaryotic peptides that mimic selectins. *J Infect Dis* 1993; **168**: 1422–1428.
12. Thomazzi SM, Sousa MHLP, Melo-Filho AA, Hewlett EL, Lima AAM, Ribeiro RA. Pertussis toxin from *Bordetella pertussis* blocks neutrophil migration and neutrophil-dependent edema in response to inflammation. *Braz J Med Biol Res* 1995; **28**: 120–124.
13. Brito GAC, Souza MHLP, Melo-Filho AA, *et al.* Role of pertussis toxin A subunit in neutrophil migration and vascular permeability. *Infect Immun* 1997; 65: 1114–1118.
14. Peumans WJ, van Damme EJM. Lectins as plant defence proteins. *Plant Physiol* 1995; **109**: 347–352.
15. Gomes JC, Ferreira RR, Cavada BS, Moreira RA, Oliveira JTA. Histamine release induced by glucose (mannose)-specific lectins isolated from Brazilian beans. Comparison with concanavalin A. *Agents Actions* 1994; **41**: 132–135.
16. Barral-Neto M, Santos SB, Barral A, *et al.* Human lymphocyte stimulation by legume lectins from the *Diocleae* tribe. *Immun Invest* 1992; **21**: 297–303.
17. Vasconcelos IM, Cavada BS, Moreira RA, Oliveira JTA. Purification and partial characterization of a lectin from the seeds of *Dioclea guianensis*. *J Food Biochem* 1991; **15**: 137–154.
18. Moreira RA, Barros ACH, Stewart JC, Pusztai A. Isolation and characterization of a lectin from the seeds of *Dioclea grandiflora* (Mart). *Planta* 1983; **158**: 63–69.
19. Oliveira JTA, Cavada BS, Moreira RA. Isolation and partial characterization of a lectin from *Cratylia floribunda* Mart. Seeds, *Revta Brasil Bot* 1991; **14**: 61–66.
20. Moreira RA, Cordeiro EF, Ramos MV, *et al.* Isolation and partial characterization of a lectin from the seeds of *Dioclea violocea* Mattius (Ex Benth). *R Bras Fisiol Veg* 1996; **8**(1): 23–30.
21. Cavada BS, Ramos MV, Cordeiro EF, *et al.* Purification and partial characterization of a lectin *Dioclea virgata* Benth seeds. *R Bras Fisiol Veg* 1996; **8**: 37–42.
22. Moreira RA, Cavada BS. Lectin from *Canavalia brasiliensis* (Mart), Isolation, characterization and behavior during germination. *Biologia Plantarum* 1984; **26**: 113–120.
23. Flores CA, Zappellini A, Prado-Franceschi J. Lipoxygenase-derived mediators may be involved in *in vivo* neutrophil migration induced by *Bothrops erythromelas* and *Bothrops alternatus* venoms. *Toxicon* 1993; **31**: 1551–1559.
24. Landucci ECT, Antunes E, Donato JL, *et al.* Inhibition of carrageenin-induced rat paw oedema by crotapotin, a polypeptide complexed with phospholipase $A_2$. *Br J Pharmcol* 1995; **114**: 578–583.
25. Moreira RA, Perroni JC. Purification and partial characterization of a lectin from *Phaseolus vulgaris*. *Plant Physiol* 1977; **59**: 783–787.
26. Rocha NP, Ferreira SH. Restoration by levamisole of endotoxin-inhibited neutrophil migration, oedema and increased vascular permeability induced by carrageenin. *Eur J Pharmacol* 1986; **122**: 87–92.
27. Braga de Mota JI, Cunha FQ, Vargaftig BB, Ferreira SH. Drug modulation of antigen-induced paw oedema in guinea-pigs: effects of lipopolysaccharide, tumour necrosis factor and leucocyte depletion. *Br J Pharmacol* 1994; **112**: 111–116.
28. Santos-de-Oliveira R, Dias-Baruffi M, Thomaz SMO, Beltramini LM, Roque-Barreira MC. A neutrophil migration-inducing lectin from *Artocarpus integrifolia*. *J Immunol* 1994; **153**: 1798–1807.
29. Nirmul G, Severin C, Taub RN. In vivo effects of concavalin A. I. *Immunusupressive effects*. *Transplantation* 1972; **14**: 91–95.
30. Mori R, Kuroda Y, Aoki T, Takenaka A, Nakamura T, Namikawa T. Supression of experimental allergic encephalomyelitis in guinea pigs and rats by concanavalin A. In: Bog-Hansen TC ed. *Lectins – Biology, Biochemistry, Clinical Biochemistry*. Berlin-New York: Walter de Gruyter, 1982; **2**: 23–32.
31. Sharon N, Lis H. Legume lectins – a large family of homologous proteins. *FASEB J* 1990; **4**: 3198–3208.
32. Sanz-Aparicio J, Hermoso J, Granjeiro TB, Calvete J, Cavada BS. The cyrstal structure of *Canavalia brasiliensis* lectin suggests a correlation between its quartenary conformation and its distinct biological properties from Concanavalin A. *FEBS Lett* 1977; 405: 114–118.
33. Lo TN, Almeida AP, Beaven MA. Dextran and carrageenan evoke different inflammatory response in rat with respect to composition of infiltrates and effect of indomethacin. *J Pharmacol Exp Ther* 1982; **221**: 261–267.
34. Hakamori S, Yasuyuki I. Functional role of glycosphingolipids in cell recognition and signaling. *J Biochem* 1995; **118**: 1091–1103.
35. Malik AB, Lo SK. Vascular endothelial adhesion molcules and tissue inflammation. *Pharmacol Rev* 1996; **48**: 213–229.
36. Cunha FQ, Poole S, Lorenzetti BB, Ferreira SH. The pivotal role of tumour necrosis factor alpha in the development of inflammatory hyperalgesia. *Br J Pharmacol* 1992; **107**: 660–664.
37. Rao TS, Currie JL, Shaffer AF, Isakson PC. In vivo characterization of zymosan-induced mouse peritoneal inflammation. *J Pharmacol Exp Ther* 1994; **269**: 917–925.
38. Patel KD, Zymmerman GA, Prescott SM, NcEver RP, McIntyre TM. Oxygen radicals induce human endothelial cells to express GMP-140 and bind neutrophils. *J Cell Biol* 1991; **12**: 749–759.
39. Guyer DA, Moore KL, Lynam EB, *et al.* P-selectin glycoprotein ligand-1 (PSGL-1) is a ligand for L-selectin in neutrophil aggregation. *Blood* 1996; **88**: 2415–2421.
40. Lasky LA, Rosen SD. The Selectins. Carbohydrate-binding adhesion molecules of the immune system. In: Gallin JI, Goldstein IM, Snyderman R eds. *Inflammation: Basic Principles and Clinical Correlates*. New York: Raven Press Ltd, 1992; 407–419.

ACKNOWLEDGEMENTS. This work was supported by CNPq, CAPES, FINEP and PADCT. Ana Maria Sampaio Assreuy thanks the Federal University of Rio de Janeiro for leave of absence to do her MSc program.

**Received 10 March 1997; accepted 1 April 1997**
